# Coexistence of t(2;14;11)(p16.1;q32;q23) and t(14;19)(q32;q13.3) chromosome translocations in a patient with chronic lymphocytic leukemia: A case report

**DOI:** 10.1097/MD.0000000000009169

**Published:** 2017-12-22

**Authors:** Guangming Liu, Zhongmei Wen, Xianglan Lu, Young Mi Kim, Xianfu Wang, Rebecca M. Crew, Mohamad A. Cherry, Shibo Li, Yuanyuan Liu

**Affiliations:** a Department of Gastroenterology; b Department of Respiratory Medicine, The First Hospital of Jilin University, Changchun, People's Republic of China; c Department of Pediatrics; d Department of Hematology, University of Oklahoma Health Sciences Center, Oklahoma City, OK.

**Keywords:** array comparative genomic hybridization, chronic lymphocytic leukemia, *MSX1* duplication, t(14 ;19), t(2 ;14 ;11)

## Abstract

**Rationale:**

With combination of multiple techniques, we have successfully characterized unique, complex chromosomal changes in a patient with chronic lymphocytic leukemia (CLL), a lymphoproliferative disorder.

**Diagnoses:**

The diagnosis was based on white blood cell, flow cytometry, and immunophenotypes and confirmed by karyotype, fluorescence in situ hybridization, and array comparative genomic hybridization from the patient's blood culture.

**Interventions:**

The patient was given fludarabine, cyclophosphamide and rituximab (FCR) for 6 cycles.

**Outcomes:**

After completion of 6 cycles of FCR, the computed tomography scans of the neck/chest/abdomen/pelvic showed that the patient in CR. During the 10-month follow-up, the patient's clinical course remained uneventful.

**Lessons:**

The translocation t(14;19) identified in this patient is a recurrent translocation found in patients with chronic B-cell lymphoproliferative disorders and the 3-way translocation involving chromosomes 2, 14, and 11 may play a role as an enhancer.

## 1 Introduction

B-cell chronic lymphocytic leukemia (CLL) is a genetically heterogeneous neoplasm characterized by the progressive accumulation of CD5+ mature B cells in bone marrow, lymph nodes, and blood. CLL is the most common leukemia in adults in Western countries and the clinical course of disease ranges from a few months of the diagnosis to ≥20 years.^[1,2]^ The frequently CLL-associated cytogenetic abnormalities include trisomy 12 (10%–20%), del11q22-q23 (5%–20%), and del13q14 (50%). In addition, del17p13 involving deletion of the tumor suppressor gene *TP53* occurs in <10% of CLL at diagnosis but up to 30% in refractory cases. Patients with del17p demonstrate aggressive diseases and have very poor prognoses.^[3–7]^

It has been reported that chromosomal translocations involving immunoglobulin heavy chain locus (*IGH)* rearrangement on 14q32 are relatively infrequent in CLL with a frequency of 4%.^[4]^ However, recent studies have revealed that translocations involving *IGH* rearrangements occur at a very high incidence rate and significantly affects survival of CLL patients.^[8–10]^ The most frequent translocation in CLL is t(14;19) (q32;q13), which juxtaposes *IGH* and *BCL3* resulting in overexpression of *BCL3*^[11]^ and is usually associated with unfavorable clinical outcome and trisomy 12.^[8,12]^ It was proposed that these 2 changes might cooperate for malignant transformation.^[13]^ In contrast, patients with del(13q) have a better survival than the patients with other cytogenetic abnormalities.^[4]^ Furthermore, t(2;14)(p13;q32) is also a recurrent chromosomal change in CLL.^[13]^

Chromosomal translocations are regarded as an important prognostic indicator and are always associated with shorter survival in B-CLL patients.^[8]^ Recently, several alternate translocations, such as t(4;14)(p16;q32) to generate *FGFR3/IGH*, t(11;14)(q13;q32) to form *CCND1/IGH*, t(14;18)(q32;q21) to produce *IGH/BCL2* fusion, and t(18;22)(q21;q11) have been identified and provided further insights into the pathogenesis of CLL.^[12–15]^ However, complex variant translocations may occur in CLL but have been rarely reported.

In the present study, as confirmed by cytogenetic analysis, we report a patient carrying the classical t(14;19)(q32;q13.3) as well as a novel 3-way translocation t(2;14;11)(p16.1;q32;q23), trisomy 12, and del(13q14). Importantly, we revealed a cryptic gain of chromosome 4p16.2 besides trisomy 12 and del(13q14.11-q21) in this patient. This study was approved by the institutional review board (IRB) at the University of Oklahoma Health Sciences Center (IRB number: 6299; Oklahoma City, OK).

## 2 Case report

### 2.1 Clinical characteristics

A 43-year-old female was admitted to the University of Oklahoma Health Sciences Center where she was diagnosed with CLL owing to weight loss and lymphadenopathy. Her hemoglobin and platelet counts were 12.8 g/dL (normal range, 12–16 g/dL) and 158 × 10^3^ cells/μL (140–440 × 10^3^ cells/μL), respectively. Her white blood cell count was 21.81 × 10^3^ cells/μL (4–11 × 10^3^ cells/μL) with relative and absolute lymphocytosis of 67% (15%–46%) and 15.7 × 10^3^ cells/μL (0.6–5.1 × 10^3^ cells/μL), respectively. Flow cytometric analysis found that her 75% monoclonal B-cells showed lambda light chain restriction of moderate intensity; her immunophenotype were as follows: CD5+, CD10-, CD19+, CD20+, CD22(dim), CD23 +/− (dim), FMC-7 +/− (dim), CD38+.

### 2.2 Cytogenetics, fluorescence in situ hybridization, and array comparative genomic hybridization analyses

**Figure 1:**
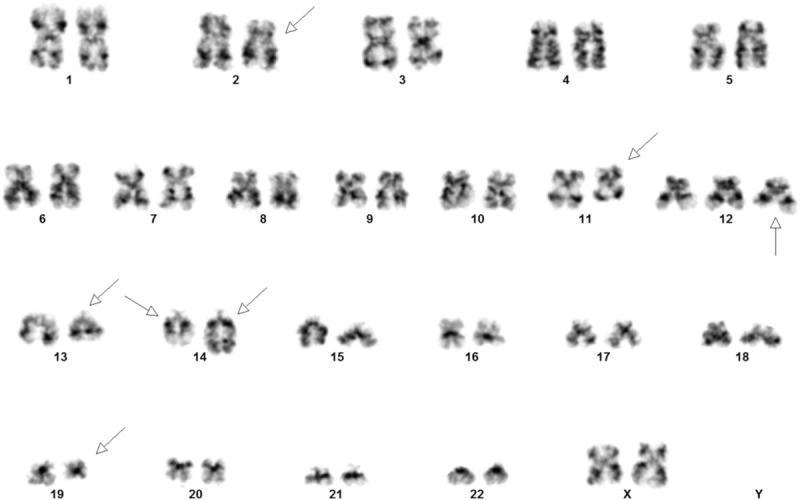
Karyogram of the clone with 2 balanced translocations, trisomy 12 and del(13q): 47,XX t(2;14;11)(p16.1;q32;q23),+12,del(13)(q14.11q21),t(14;19) (q32;q13.3).

At diagnosis, the patient's B-cells were subjected to karyotype analyses. The results revealed that 25% (5/20) of the metaphase chromosome displayed a variant translocation among chromosomes 2, 11, and 14, and a translocation between 2 chromosomes 14 and 19 as well as +12 and del(13)(q14.11-q21). The karyotype was designated as 47, XX, t(2;14;11) (p16.1;q32;q23), +12, del(13)(q14.11q21), t(14;19) (q32;q13.3) (Fig. 1).

Fluorescence in situ hybridization (FISH) analyses were performed in the uncultured and cultured cells using the *LSI IGH* and *LSI MLL* dual color break-apart rearrangement probes (Abbott Molecular, Inc., Des Plaines, IL) and *LSI BCL11A* and *LSI BCL3* dual color break-apart rearrangement probes (Empire Genomics, Inc., Buffalo, NY). The uncultured cells were also tested using CLL panel (Abbott Molecular, Inc., Des Plaines, IL). All the experimental procedures followed the manufacturers’ instructions.

**Figure 2:**
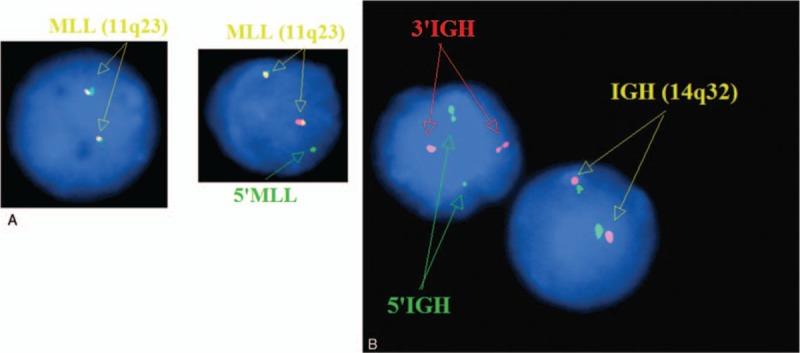
Interphase FISH analysis. (A) Interphase FISH by DNA-probe *LSI MLL* (Abbott) using a Spectrum Green–labeled on *5’MLL* and Spectrum Red–labeled on *3’MLL*. One green and 1 yellow signal (including 1 tiny green signal and 1 normal red signal) indicated variant *MLL* gene break-apart on the right side, Normal FISH signal is shown on the left side of the panel for comparison. (B) Interphase FISH by DNA-probe *LSI IGH* (Abbott) using a Spectrum Green–labeled on *5’IGH* and Spectrum Red–labeled on *3’IGH*, 2 red and 2 green signals indicated the rearrangement of biallelic *IGH*, and 2 yellow signals indicated the normal *IGH*. FISH = fluorescence in situ hybridization.

On uncultured interphase cells, FISH did not reveal t(11;14)(q13;q32), t(14;18)(q32;q21), del(6)(q23), del(11)(q22), del(17)(p13), but found trisomy 12 in 74% and monoallelic 13q14 deletion in 21% of tested cells. Moreover, variant *MLL* gene break-apart signals in 16% of 200 examined cells were observed as demonstrated by 1 tiny Spectrum Green signal and 1 normal Spectrum Red signal in Figure 2A, indicating the *MLL* gene break-apart. Furthermore, 104 of 200 cells (52%) exhibited a biallelic rearrangement of *IGH* (14q32) as showed by Spectrum Red and Spectrum Green signals when compared to Spectrum Orange signals in the normal *IGH* (Fig. 2B).

**Figure 3:**
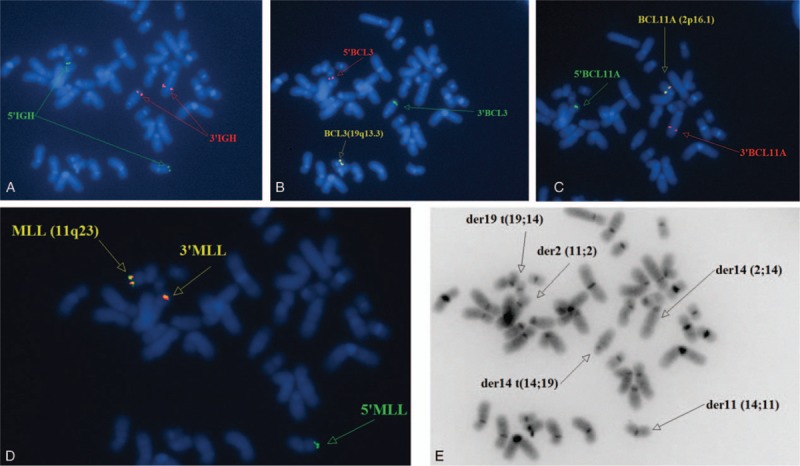
Metaphase FISH analysis. (A) Metaphase FISH by DNA-probe *LSI IGH* (Abbott) using a Spectrum Green–labeled on *5’IGH* and Spectrum Red–labeled on *3’IGH* indicated biallelic *IGH* rearrangement. Small translocated segments of *5’IGH* is on der 11 and der19. (B) Metaphase FISH by DNA-probe *LSI BCL3* (Empire Genomics) using a Spectrum Green–labeled on *3’BCL3* and Spectrum Red–labeled on *5’BCL3* indicated *BCL3* rearrangement. Small translocated segment of *3’BCL3* is on der 14. (C) Metaphase FISH by DNA-probe *LSI BCL11A* (Empire Genomics) using a Spectrum Green–labeled on *5’BCL11A* and Spectrum Red–labeled on *3’BCL11A* indicated *BCL11A* rearrangement. Small translocated segment of *3’BCL11A* is on der 14. (D) Metaphase FISH by DNA-probe *LSI MLL* (Abbott) using a Spectrum Green–labeled on *5’MLL* and Spectrum Orange–labeled on *3’MLL* indicated variant *MLL* rearrangement. Small translocated segments of *3’MLL*(including 1 tiny green signal and 1 normal red signal) is on der 2. (E) Summary of the metaphase by FISH analysis of bone marrow. FISH = fluorescence in situ hybridization.

Analyses of karyotyping and FISH results from metaphase cells showed that *3’IGH* signals were located on both der(14) and *5’IGH* were translocated to der(11) and der(19), respectively (Fig. 3A); *5’BCL3* signal was located on der(19) and *3’BCL3* was translocated to der(14) (Fig. 3B); *5’BCL11A* signal was located on der(2) and *3’BCL11A* was translocated to der(14) (Fig. 3C); *5’MLL* signal was located on der(11) and *3’MLL* was translocated to der(2) (Fig. 3D). Taken together, these analyses in metaphase cells confirmed the complex translocations among chromosomes 2, 11, and 14 as well as chromosomes 14 and 19 (Fig. 3E).

**Figure 4:**
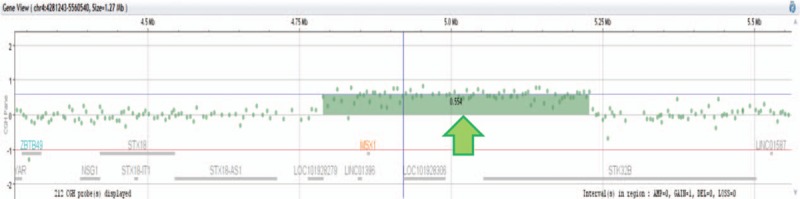
Results of the oligoarray CGH using NimbleGen SegMNT and the RefSeq genes in the abnormal region (University of California, Santa Cruz genome browser hg19). The *y* axis indicates a gain or loss of genetic material, while the *x* axis indicates the genomic position of each feature on the chromosome. The green arrow indicates gain and the significant gene in the gained region is listed. Gain of 4p16.2 (4,788,290–5,227,609 bp, hg19; ∼0.4 Mb). CGH = array comparative genomic hybridization.

Further array comparative genomic hybridization (CGH) analyses on the patient's DNA sample revealed the presence of an extra chromosome 12 and deletion of 13q14.11-q21, which was consistent with our karyotype analyses. Interestingly, we also found a gain of 4p16.2 (4,788,290–5,227,609 bp hg19; 0.4 Mb) containing *MSX1* (msh homeobox 1) gene (Fig. 4), which plays a pivotal role in early hematopoietic development and malignancy transformation.

## 3 Discussion

The case reported here is unique and particularly interesting, as 2 balanced translocations including t(14;19)(q32;q13.3)/*IGH;BCL3* and t(2;14;11) (p16.1;q32;q23)/*BCL11A;IGH;MLL* were observed simultaneously in one patient in addition to trisomy 12 and del(13q). In addition, we detected a microduplication in 4p16.2 involving the *MSX1* gene that plays an important role during early hematopoiesis. Importantly, we found a biallelic rearrangement of *IGH* (14q32) with partner genes *BCL11A*, *MLL,* and *BCL3* in our case.

Translocations involving *IGH* not only promote pathogenesis but also predict a poor prognosis of B-cell malignancies such as t(8;14)(q24;q32)(*MYC/IGH*) in Burkitt lymphoma (BL); t(11;14)(q13;q32) (*CCND1/IGH*) in mantle cell lymphoma (MCL); and t(14;18)(q32;q21) (*IGH/BCL2*) in follicular lymphoma (FL).^[8]^ The cases with concomitant t(2;14) and t(14;19) translocations have previously been reported,^[16]^ and they are always regarded as a rare recurrent chromosomal changes associated with atypical cytology, trisomy 12, and a progressive disease in CLL.^[13,17,18]^

The present study is the first report of a CLL case with a complex variant translocation involving 3 chromosomes 2, 11, 14 named t(2;14;11)(p16.1;q32;q23)/*BCL11A;IGH;MLL*, especially concurrent with t(14;19)(q32;q13.3)/*IGH;BCL3.* In contrast to *MYC/IGH, CCND1/IGH* and *IGH/BCL2*, the roles for *IGH;BCL3* and *BCL11A;IGH;MLL* fusions in CLL remain poorly understood; however, it is possible the target genes that become overexpressed or gained new functions may be relevant to the poor prognosis of CLL.

The *MLL* gene rearrangement often occurs in acute myelocytic leukemia (AML), acute lymphoblastic leukemia, and myelodysplastic syndrome. In hematologic malignancies such as CLL, the most common abnormality is the deletion of the *MLL(11q23)*,^[19]^ whereas the *MLL* gene rearrangement has not been previously observed in CLL. In this study, we revealed this interesting 3-way translocation of the *MLL* gene rearrangement; whether it contributes to the leukemia progression or even an unfavorable prognosis in CLL warrants further investigation.

It is widely believed that presence of only the *IGH* rearrangement is not sufficient to induce tumorigenesis, and acquisition of additional genetic aberrations is necessary for malignant transformation.^[8]^ Trisomy 12, observed in this case, is one such genetic anomaly. The cytogenetic abnormality of trisomy 12 associated with intermediate prognosis is observed in up to 50% of *IGH/BCL3*-positive B-CLLs and was considered to act cooperatively with t(14;19) in leukemogenesis.^[20]^ Interestingly, however, it was reported that patients with 13q deletions as a sole abnormality had the longest estimated survival times compared with other cytogenetic abnormalities.^[4,21]^ Moreover, *miR-15a* and *miR-16–1* locate in this region, and negatively regulate *BCL2* expression at a posttranscriptional level.^[22]^

*MSX1* was found to be overexpressed in cell lines derived from MCL and leukemia AML as well as in 3% of patients with MCL and AML.^[23]^ In the present study, array CGH revealed a cryptic gain of *MSX1* gene besides trisomy 12 and del(13q14.11-q21), which has not been reported previously in CLL. These data suggest an oncogenic role for *MSX1* in leukemogenesis.

In summary, we reported a rare case of an adult CLL patient with the coexistence of classical *IGH/BCL3* translocation and a three-way variant translocation *BCL11A/IGH/MLL*, as well as trisomy 12 and del(13q). Furthermore, a cryptic genomic alteration involving leukemia-related *MSX1* gene was found in this case at the level of the array CGH.
